# Acetate reprograms gut microbiota during alcohol consumption

**DOI:** 10.1038/s41467-022-31973-2

**Published:** 2022-08-08

**Authors:** Cameron Martino, Livia S. Zaramela, Bei Gao, Mallory Embree, Janna Tarasova, Seth J. Parker, Yanhan Wang, Huikuan Chu, Peng Chen, Kuei-Chuan Lee, Daniela Domingos Galzerani, Jivani M. Gengatharan, Asama Lekbua, Maxwell Neal, Rob Knight, Hidekazu Tsukamoto, Christian M. Metallo, Bernd Schnabl, Karsten Zengler

**Affiliations:** 1 Department of Pediatrics, University of California San Diego, La Jolla, CA USA; 2 Center for Microbiome Innovation, University of California San Diego, La Jolla, CA USA; 3 Bioinformatics and Systems Biology Program, University of California San Diego, La Jolla, CA USA; 4 Department of Medicine, University of California San Diego, La Jolla, CA USA; 5 Department of Bioengineering, University of California, San Diego, CA USA; 6 Molecular and Cellular Biology Laboratory, The Salk Institute for Biological Studies, La Jolla, CA USA; 7 Department of Computer Science and Engineering, University of California San Diego, La Jolla, CA USA; 8 Southern California Research Center for ALPD and Cirrhosis and Department of Pathology, La Jolla, CA USA; 9 Department of Veterans Affairs Greater Los Angeles Healthcare System, Los Angeles, CA USA; 10 Department of Medicine, VA San Diego Healthcare System, San Diego, CA USA

**Keywords:** Microbiome, Metagenomics

## Abstract

Liver damage due to chronic alcohol use is among the most prevalent liver diseases. Alcohol consumption frequency is a strong factor of microbiota variance. Here we use isotope labeled [1-^13^C] ethanol, metagenomics, and metatranscriptomics in ethanol-feeding and intragastric mouse models to investigate the metabolic impacts of alcohol consumption on the gut microbiota. First, we show that although stable isotope labeled [1-^13^C] ethanol contributes to fatty acid pools in the liver, plasma, and cecum contents of mice, there is no evidence of ethanol metabolism by gut microbiota ex vivo under anaerobic conditions. Next, we observe through metatranscriptomics that the gut microbiota responds to ethanol-feeding by activating acetate dissimilation, not by metabolizing ethanol directly. We demonstrate that blood acetate concentrations are elevated during ethanol consumption. Finally, by increasing systemic acetate levels with glyceryl triacetate supplementation, we do not observe any impact on liver disease, but do induce similar gut microbiota alterations as chronic ethanol-feeding in mice. Our results show that ethanol is not directly metabolized by the gut microbiota, and changes in the gut microbiota linked to ethanol are a side effect of elevated acetate levels. De-trending for these acetate effects may be critical for understanding gut microbiota changes that cause alcohol-related liver disease.

## Abstract (web summary)

Chronic alcohol use is associated with intestinal bacterial overgrowth and dysbiosis, but the contribution of ethanol is unclear. Here, using mouse models, the authors find that ethanol is not directly metabolized by the gut microbiota, and that induced changes are rather a side effect of elevated acetate levels.

## Introduction

Alcohol-related liver disease is among the most prevalent liver diseases in the United States and Europe^1,2^. Excessive alcohol consumption causes a range of liver injuries, progressing from steatosis, to steatohepatitis, fibrosis, and ultimately cirrhosis. During alcohol consumption, alcohol is rapidly absorbed by diffusion, mainly in the upper gastrointestinal tract and then enters the liver via the portal vein. The effect of alcohol on the distal small intestine and colon largely comes from circulatory alcohol during the equilibration process between the lumen of the gastrointestinal tract and vascular space^3^. Alcohol consumption has been shown to alter the stool microbiota composition^4,5^ and function^6^, but how relatively small concentrations of ethanol in the large intestine cause the profound changes of the stool microbiota with which they have been associated is currently poorly understood.

Ethanol is predominantly metabolized in hepatocytes, where alcohol dehydrogenase converts it to acetaldehyde, then acetaldehyde dehydrogenase further metabolizes it to acetate. Acetaldehyde dehydrogenase plays a key role in determining peripheral acetaldehyde levels. A systemic increase of acetate, but not acetaldehyde, has been observed after alcohol intake in humans^7,8^. Ethanol can also be metabolized into acetaldehyde by cytochrome P450, family 2, subfamily e, polypeptide 1 (Cyp2e1), associated with production of radical oxygen species and lipid peroxidation^9^. Although most alcohol metabolism occurs in hepatocytes, the intestinal mucosa also expresses enzymes involved in oxidative metabolism of alcohol^10^. This is of particular interest, as we recently demonstrated that ethanol-feeding increases intestinal acetate levels^11^.

The gastrointestinal tract is the habitat for the gut microbiota. Gut microbiota alterations and bacterial overgrowth have been observed after alcohol consumption^4,12,13^. However, the mechanisms by which ethanol alters microbial composition are not clear. Chronic alcohol consumption is associated with lower intestinal expression of antimicrobial molecules, such as regenerating islet-derived (Reg)−3 lectins. We tested whether ethanol regulated the gut microbiota through Reg3g in our previous study. However, we found no significant differences in the luminal gut microbiota composition between the ethanol-fed *Reg3g* deficient and wild-type littermate mice^14^, suggesting that gut microbiota alteration induced by ethanol is not regulated via Reg3g.

Patients with alcohol-related liver disease have altered gut microbiota^4–6,15–23^. Intestinal bacterial overgrowth is also commonly found in patients with alcohol-related liver disease^24–27^. Establishing causality, gavage of fecal samples from alcoholic hepatitis patients but not controls increased susceptibility of initially germ-free mice to ethanol-induced liver disease^20^. Fecal microbiota transplantation from healthy subjects to severe steroid-ineligible patients with alcoholic hepatitis improved patient survival, suggesting that redressing microbiome alterations induced by alcohol consumption could be beneficial^28^. Although these studies indicate that the gut microbiota acts as a key player in alcohol-related liver disease, they do not address whether the gut microbiota can directly metabolize ethanol. Ethanol is rapidly absorbed in the stomach and upper small intestine. Intraluminal ethanol concentrations in the ileum following alcohol ingestion are similar to serum ethanol levels in humans, indicating a continuous equilibration between the gastrointestinal tract and vascular space rather than ethanol traveling the length of the intestine^3^. Chronic ethanol ingestion accelerated chemically induced rectal carcinogenesis, and the authors suggested that during this process, acetaldehyde possibly generated through bacterial ethanol oxidation might play a role^29^, although they did not establish this causal link. Understanding whether the gut microbiome directly metabolizes ethanol, and whether changes in the gut microbiome are related to ethanol consumption per se are critical for moving beyond microbiome associations and towards identifying bacteria that are causal for deleterious effects of alcohol consumption, rather than side-effects either of consumption or disease. A strong parallel is early studies of the microbiome in diabetes, where many microbiome changes attributed to diabetes per se were actually effects of metformin treatment for diabetes, so suppressing these differences between diabetic patients and controls would actually have had a counterproductive effect^30^.

In this work, we integrate shotgun metagenomics, metatranscriptomics, and targeted metabolomics to show that alterations in the gut microbial community during ethanol consumption is linked to elevated acetate levels and not by ethanol being directly metabolized by the gut microbiota. Moreover, by increasing systemic acetate levels in mice, we show the gut microbial community compositions are similar to ethanol consumption, but increased acetate alone does not induce liver damage.

## Results

### Contribution of ethanol to acetate and acetyl coenzyme A (acetyl-CoA) pool

**Fig. 1:**
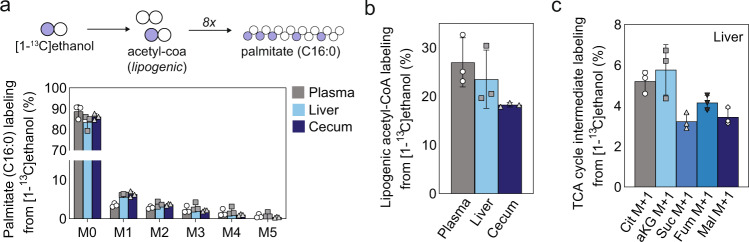
Contribution of ethanol to acetate and acetyl CoA pool. **a** Palmitate (C16:0) labeling from [1-^13^C] ethanol (y-axis), Mn represents the nth isotopologue in the mass isotope distribution (MID) for palmitate (C16:0), the most abundant fatty acid (x-axis). **b** Lipogenic acetyl CoA labeling from [1-^13^C] ethanol (*N* = 3). **c** TCA cycle intermediate labeling from [1-^13^C] ethanol (*N* = 3). Cit citrate, αKG α-ketoglutarate, Suc succinate, Fum fumarate, Mal malate. Bar plots represent the mean value and the error bars the standard error. Source data are provided as a Source Data file.

To investigate ethanol metabolism and trace the fate of ethanol, we administered stable isotope labeled [1-^13^C] ethanol to mice via oral gavage. Isotope enrichment in palmitate isolated from plasma, liver, and cecum contents was analyzed by isotopomer spectral analysis (Fig. 1a). Ethanol contributed to acetate and acetyl-CoA pools, with up to 27%, 24%, and 18% of lipid carbon in newly synthesized fatty acids arising from ethanol in plasma, liver, and cecum, respectively (Fig. 1b). Further, we examined the contribution of [1-^13^C] ethanol to intermediates of the TCA cycle in the liver, including citrate, α-ketoglutarate, succinate, fumarate, and malate. We found these polar metabolites were only slightly enriched (3–6%) in liver samples (Fig. 1c). In addition, acetyl-CoA carboxylase (ACC) inhibitor ND646 decreased palmitate synthesis to approximately ~0.5% in plasma, liver, and cecum (Supplementary Fig. 1). These results indicate that oral administration of [1-^13^C] ethanol is contributing to the acetate and acetyl-CoA pools, and is mainly used for de novo lipogenesis.

### Ethanol is not directly metabolized by the gut microbiota ex vivo

To explore if gut microbiota could directly metabolize ethanol and contribute to the acetate pool, we incubated cecal contents from mice treated with or without oral gavage of ethanol (*N* = 6), in anoxic medium containing ethanol or glucose (*N* = 12). While anoxic oxidation of ethanol to acetate is thermodynamically not favorable (Supplementary Table 1), we reasoned that ethanol could theoretically be co-metabolized in the presence of other organics in the cecal content^31^. We found that ethanol was not toxic to the cells since we observed dense growth of microorganisms, which was likely due to residual organics present in intestinal contents (Supplementary Fig. 2a). However, cultures with ethanol had no growth and ethanol was not consumed during 96 h of incubation (Supplementary Fig. 2b). To investigate the ability of gut microbes to produce short chain fatty acids (SCFAs) from ethanol, we measured eight common SCFAs (acetate, butyrate, caproate, heptanoate, isobutyrate, isovalerate, propionate, and valerate) at time 0 and after 72 h of growth. The production of acetate was not significantly different in ex vivo cultures with or without ethanol. Variations in the concentration of other SCFAs were also not significantly different (<1 µM), which is likely due to residual organics present in intestinal contents (Supplementary Fig. 2c). These results indicate that ethanol could not be directly metabolized into SCFAs by the gut microbiota ex vivo.

### Microbial alcohol dehydrogenase is not upregulated by ethanol-feeding in mice

**Fig. 2:**
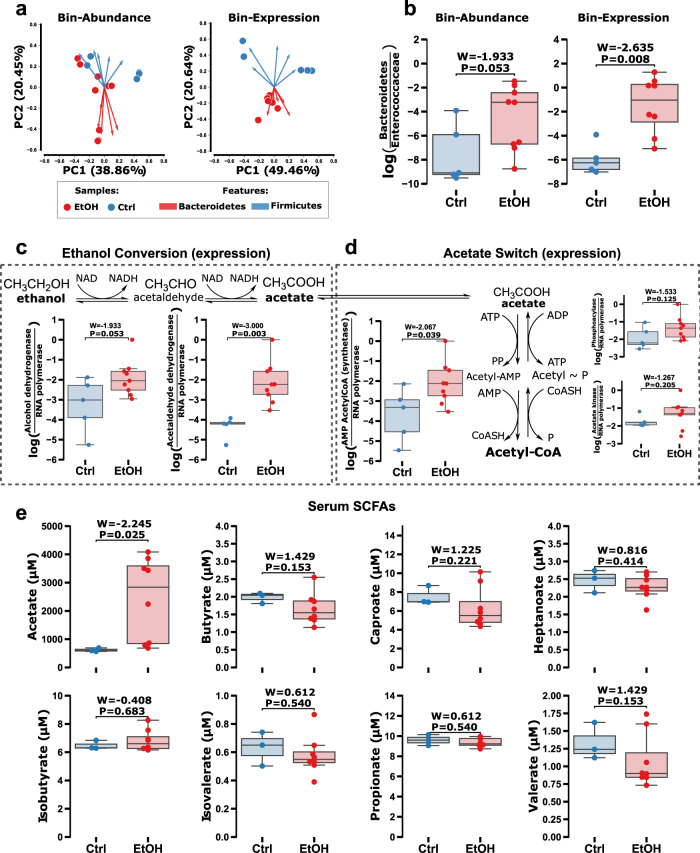
Acetate scavenging and Bacteroides strains are upregulated while alcohol dehydrogenation is not changed in alcohol (red) fed mice compared to controls (blue). **a** Compositional biplot of Aitchison distances on bin abundance (left) and expression (right) between conditions with arrows colored by Phylum level taxonomy. **b** The log-ratio of Bacteroidetes and Enterococcaceae strains (y-axes) identified in biplot for bin abundance (left) and expression (right) compared between treatment groups (x-axes). **c** Microbial alcohol dehydrogenase (left) and acetaldehyde dehydrogenase (right) log-ratios compared between treatments (x-axes). **d** Conversion in acetate switch for dissimilation (left) or excretion (right) log-ratios (y-axes) compared between treatments (x-axes). Serum short-chain fatty acids (SCFAs) (y-axis) after ethanol-feeding (x-axes) for (**e**) acetate, (**e** top left) butyrate, (**e** bottom left) propionate, (**e** top right) isovalerate, and (**e** bottom right) valerate. Box plots represent the minimum, maximum, median, first, and third quartile values (shaded region). Significance was evaluated by a two-sided Wilcoxon rank sum test with Tukey’s HSD post-hoc test adjusted *p* values of less than 0.05 were shown in the figure (*N* = 14). Source data are provided as a Source Data file.

Next, we evaluated if the gut microbiota could directly metabolize ethanol and contribute to the acetate pool in vivo. We interrogated the response of the gut microbiota to oral gavage ethanol-feeding in a mouse model through shotgun metagenomic and metatranscriptomics sequencing of the cecum material. Sequence reads were co-assembled, genomes binned (bin), functional genes predicted, abundance and transcription tables generated (see Methods). After ethanol-feeding, the gut microbiota was altered as shown in the clustering through the Robust Principal component analysis (RPCA)^32^ compositional biplot and Aitchison beta-diversity distance on both bin abundance (*N* = 14, Permutational multivariate analysis of variance (PERMANOVA)^33^; Pseudo-F = 2.95, *P* = 0.003) and transcription (*N* = 14, PERMANOVA; Pseudo-F = 2.47, *P* = 0.004). Ethanol-fed mice and control mice were differentiated by the phyla Bacteroidetes and Firmicutes as revealed by both bin abundance, obtained by metagenomics as described below (Fig. 2a left) and expression (Fig. 2a right). The log-ratio was significant for both the increased abundance and overall expression levels of Bacteroidetes relative to Enterococcaceae in ethanol-fed mice (Fig. 2b).

The log-ratio of microbial alcohol dehydrogenase gene expression relative to the rpoA housekeeping gene in cecum samples was not significantly altered in the ethanol-fed mice (Fig. 2c). Next, we explored the log-ratios of acetate scavenging gene expression relative to the rpoA housekeeping gene. The log-ratio of AMP acetyl-CoA synthetase gene expression, which is involved in acetate dissimilation to acetyl-CoA, was significantly upregulated in ethanol-fed mice compared to controls. However, the log-ratio was not significantly altered for phosphoacylase and acetate kinase gene expression, which are involved in acetate excretion (Fig. 2d). These results indicate that instead of metabolizing ethanol directly via alcohol dehydrogenase, the gut microbiota were responding to ethanol-feeding through acetate dissimilation. The serum acetate concentration was significantly increased (4X increase) in ethanol-fed mice compared with controls. Increased serum acetate during alcohol consumption from ethanol broken down in the liver has been observed previously in human subjects^8^. Other SCFAs (butyrate, caproate, heptanoate, isobutyrate, isovalerate, propionate, and valerate) were not significantly altered in the serum samples (Fig. 2e).

**Fig. 3:**
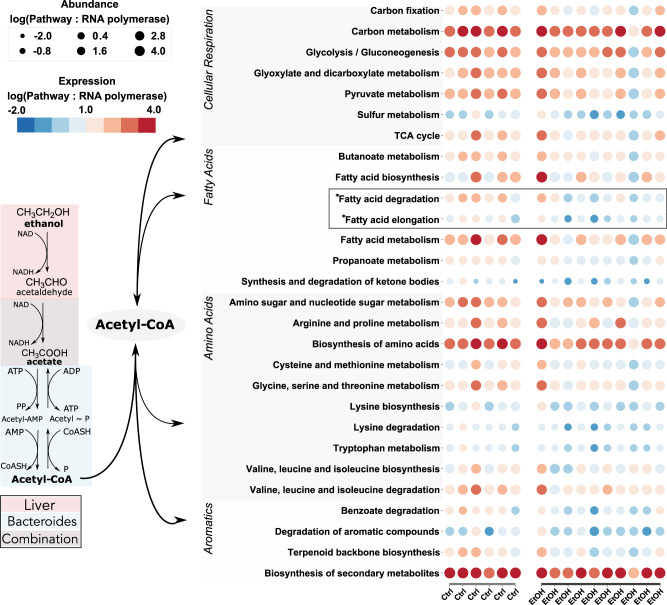
Acetate and acetaldehyde converted from ethanol in the liver converted to acetyl-CoA by *Bacteroides spp*. are used in gluconeogenesis. (left) Pathways of ethanol to acetate (liver; red) and acetate/acetaldehyde conversion to acetyl-CoA (species of the Bacteroidetes phylum; blue). (right) Expression (color bar) and abundance (dot size) for pathways involved in the excretion or dissimilation of acetyl-CoA. The Gluconeogenesis pathway (bold) is upregulated in alcohol treatment compared to controls. Source data are provided as a Source Data file.

The log-ratio of acetaldehyde dehydrogenase gene expression relative to the rpoA housekeeping gene was also increased in ethanol-fed mice (Fig. 2c). This suggested that acetaldehyde converted from ethanol by the host^8^, is further oxidized to acetate by the gut microbiota. Acetate to acetyl CoA genes were exclusively detected in bins of the phylum Bacteroidetes (Supplementary Table 2). In line with the upregulation of microbial acetaldehyde dehydrogenase and AMP acetyl-CoA synthetase gene expression, gluconeogenesis was also upregulated by Bacteroidetes bins in ethanol-fed mice (Fig. 3). Similar to ethanol, the anaerobic metabolism of exogenously derived acetate is not thermodynamically favorable alone but is feasible through co-metabolism which is commonly found in the gut microbiome^34–36^. Many of the bins with transcription of genes required to metabolize acetate (bin.135, bin.480, bin.412) fell within the class Bacteroidia, in particular, bin.135 in the genus of *Bacteroides*, which has been previously characterized as enriched during consumption of alcohol (Supplementary Fig. 3, Supplementary Table 2)^37^. Based on this we hypothesized that *Bacteroides spp*. may utilize exogenous acetate in co-metabolism with organics. To determine which combination to test we utilized a genome-scale metabolic model (GEM) of *Bacteroides fragilis* which predicted co-metabolism of acetate in the presence of methionine or glycerol (see Methods). To test this hypothesis, we cultivated *Bacteroides fragilis* anaerobically in Clostridial Basal media^38^ with or without acetate, glycerol, methionine, or glucose. We observed growth similar to that of glucose alone with the addition of glycerol and acetate but not acetate and/or methionine alone (Supplementary Fig. 4). This demonstrates that *Bacteroides* species may play a role in the metabolism of exogenous acetate pools in the gut originating from the conversion of ethanol to acetate in the liver, providing one possible explanation of their increased abundance with alcohol consumption.

### Acetate mimics gut microbiota alterations induced by ethanol-feeding

**Fig. 4:**
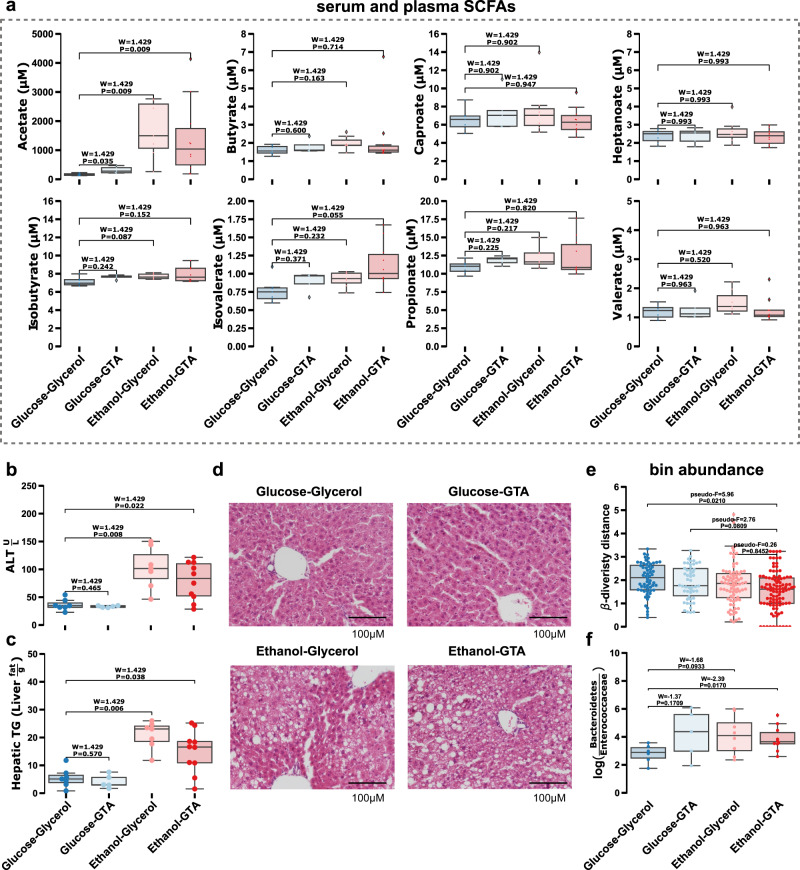
Treatment of glyceryl triacetate (GTA) with or without alcohol artificially enriches blood acetate and mimics microbial changes observed in ethanol only treatment but does not damage the liver. **a** Whole blood Short Chain Fatty Acid (SCFA) measurements, **b** serum alanine transaminase (ALT), **c** hepatic triglyceride (TG) liver damage measurements, and **d** representative sections of the liver after hematoxylin and eosin staining between intragastric feeding model of continuous infusion of ethanol or glucose with or without GTA in mice (*N* = 4). Scale bar, 100 µM. **e** Beta-diversity distance from Ethanol-GTA treatment group compared between treatment groups. *p* value determined by pairwise PERMANOVA between treatment groups. **f** The log-ratio of Bacteroides and Enterococcaceae (y-axis) compared between treatments (x-axis). Box plots represent the minimum, maximum, median, first, and third quartile values (shaded region). Significance was evaluated by a two-sided Wilcoxon rank sum test with Tukey’s HSD post-hoc test adjusted *p* values of less than 0.05 were shown in the figure (*N* = 29). Source data are provided as a Source Data file.

To validate these observations, mice were subjected to an intragastric ethanol-feeding model and supplemented with GTA (1.0 g/kg body weight) or glycerol as control and compared to mice that were fed with glucose instead of ethanol (plus/minus GTA supplementation) (*N* = 29). The GTA dosage was determined, through a preceding experiment with levels of GTA at low (0.1 g/kg body weight), medium (1.0 g/kg body weight), and high (6.0 g/kg body weight) doses tested over time (*N* = 4). The medium dose was chosen for the larger experiment due to exhibiting significantly different blood acetate levels (Supplementary Fig. 5). In the subsequent larger experiment (*N* = 29), blood acetate levels were significantly increased in the ethanol-glycerol, ethanol-GTA, and glucose-GTA treatment groups compared to those mice in the glucose-glycerol group. We found no significant changes of other SCFAs in these experiments (Fig. 4a). Without ethanol, liver damage was not observed in GTA treatment groups, as assessed by serum alanine transaminase (ALT), hepatic triglyceride (TG) level, and hepatic steatosis (Fig. 4b–d, Supplementary Fig. 6), suggesting that ethanol-induced liver disease was not altered by GTA supplementation. The microbiome composition evaluated by shotgun metagenomics and subsequent analysis through Aitchison beta-diversity distances from ethanol-fed mice treated with GTA were significant for only the glucose-fed mice treated without GTA. This suggests that the microbiome of mice with increased blood acetate levels (achieved by GTA supplementation) was more similar to ethanol-fed groups (Fig. 4e). A Bacteroidetes enrichment relative to Enterococcaceae was observed in the GTA only (glucose-GTA) treatment group, mimicking that of the ethanol only (ethanol-glycerol) treatment group (Fig. 4f).

The absorption and hepatic metabolism of ethanol was not significantly altered in ethanol-fed mice treated with GTA, as demonstrated by plasma ethanol level (Supplementary Fig. 6A), hepatic alcohol dehydrogenase (Adh) activity (Supplementary Fig. 6B), and protein expression of Cyp2e1 (Supplementary Fig. 6C, Supplementary Fig. 7). GTA treatment did not affect intestinal permeability compared with glycerol treatment following chronic ethanol-feeding, as assessed by fecal albumin level (Supplementary Fig. 6D). Taken together, increasing blood acetate levels alone induces similar changes in the cecum microbiota to those induced by chronic ethanol-feeding. Acetate-associated gut microbiota changes are not sufficient to induce liver disease in the absence of ethanol.

## Discussion

**Fig. 5:**
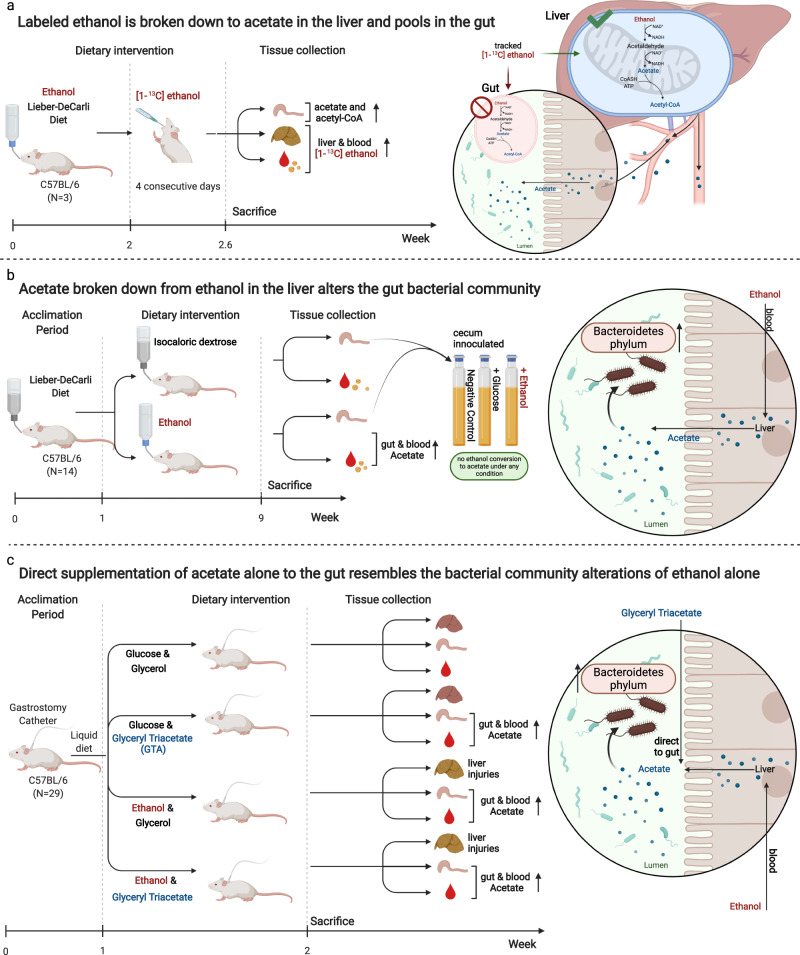
Summary of three main experiments. **a** Experiment one utilized oral gavage of labeled ethanol, in order to evaluate where it was being metabolized. From this, we found that ethanol is broken down in the liver to acetate which is then released into circulation with an increased pool forming in the gut. **b** Experiment two consistent of two stages. First, mice were fed a Lieber DeCarli diet for a total of 9 weeks after which the blood short chain fatty acids (SCFA) along with abundance and transcription of cecum microbiota were compared between conditions. Second, the cecum microbiota were collected and grown anaerobically in minimal media with or without ethanol. These experiments showed that anaerobic gut bacteria, in particular species of the phylum Bacteroidetes, do not break down ethanol to acetate but rather utilize acetate produced from the liver for gluconeogenesis. **c** Experiment three replicated acetate levels found in the gut during oral gavage of ethanol through the intragastric infusion of Glyceryl Triacetate (GTA) which increases gut acetate levels but not blood. For comparison, four conditions were performed being glucose vs. ethanol and glycerol vs. GTA in combination. The gut microbiota abundance, liver damage, blood SCFA, and gut acetate levels were measured. This experiment demonstrated that GTA causes similar alterations in the gut microbiome to that of ethanol, with increases in the phylum Bacteroidetes, but did not cause liver damage. Created with BioRender.com.

Alcohol-related liver disease (ALD) is associated with profound changes in the gut microbiota including the alterations of Bacteroidetes and Enterococcaceae^6,17,18^. However, shift in Bacteroidetes and Enterococcaceae abundance have been associated with many other host phenotypes^39^ and little is known about the mechanisms by which ethanol alters microbial composition. Using metatranscriptomics, we found that microbial alcohol dehydrogenation was not significantly altered following ethanol-feeding, but AMP acetyl-CoA synthetase gene expression was upregulated. This interesting finding suggested that microbial ethanol metabolism does not contribute significantly to observed changes in the gut microbiota and that these changes might be induced via acetate. Therefore, we tested our hypothesis using an intragastric ethanol-feeding mouse model by supplementing GTA. We showed that GTA supplementation increased blood acetate levels, mimicked the gut microbiota changes observed with ethanol-feeding, but did not induce liver damage in the absence of alcohol. These findings indicate that acetate-induced alterations of the gut microbiota alone without alcohol are not sufficient to induce liver damage. Supplementation with acetate did not protect the liver from ethanol-induced liver injury either, although supplementation of another short chain fatty acid, butyrate, did reduce ethanol-induced liver damage^40^ (Fig. 5).

Although high-alcohol-producing bacteria were identified in humans^41^, whether alcohol-metabolizing strains exist in humans is not clear. Following acute administration of ethanol to germ-free and conventional rates, acetaldehyde concentrations were significantly lower in the rectum and cecum in germ-free mice compared with conventional animals, and this was paralleled by bacteria numbers in the two intestinal locations, suggesting that acetaldehyde could be generated by bacterial ethanol oxidation^29^. However, our study provides no evidence that gut microbiota can metabolize ethanol directly, as demonstrated by ex vivo and in vivo experiments. Significant ethanol consumption was not detected ex vivo. Microbial alcohol dehydrogenase gene expression was not upregulated after ethanol-feeding in mice. In contrast, the relative expression of a microbial gene involved in acetate dissimilation, AMP acetyl-CoA synthetase, was significantly upregulated in alcohol-fed mice relative to controls. We demonstrate here that the gut microbiome altered by acetate and not ethanol, do not play a role in liver damage. Conventional mice exert relatively mild liver disease. Following microbiota transplantation with stool from patients with severe alcohol-associated liver disease, severity of ethanol-induced liver disease increases in microbiota-humanized mice^20^. Virulence factors in pathobionts and pathogens are important determinants of liver disease in microbiota-humanized mice and patients^16^, and they are likely independent from acetate. Moreover, we did not explore the possible role of the oral microbiota in the metabolism of ethanol, where potential bacterial community members have been shown to metabolize ethanol to acetaldehyde in culture^42^. Future work will be needed to address the role of these oral microbiota species in vivo, the possible role of bile acids, and to replicate these findings in larger human cohorts^22^.

Acetate metabolism confers various metabolic functions, such as energy production, lipid synthesis and protein acetylation^43^. The “acetate switch” occurs when cells deplete acetate-producing carbon sources and begin to scavenge for environmental acetate to succeed^44^. Alcohol-induced elevation in acetate blood levels has been observed in humans^8^. In our study, blood acetate level was significantly increased in ethanol-fed mice compared with controls in the oral gavage and alcoholic hepatitis mouse models. The increase of the serum acetate might lead to the increase of acetate in the intestine where the gut microbiota resides. To adapt to the new environment with plenty of acetate available, the gut microbiota imports and utilizes acetate. After being imported into the microbial cells, acetate dissimilation is activated and acetate is used for the production of acetyl-CoA, which enters TCA cycle, gluconeogenesis, glyoxylate cycle and de novo fatty acid biosynthesis. Especially, gluconeogenesis was upregulated in the alcohol treatment groups compared with controls. In contrast, microbial gene expressions involved in acetate excretion including phosphoacylase and acetate kinase, were not significantly different between ethanol-fed mice and controls. These results suggested that ethanol-feeding activated the acetate dissimilation process in the gut microbiota.

In summary, our study showed that ethanol is not directly metabolized by the gut microbiota. The gut microbiota responds to ethanol-feeding through acetate dissimilation and GTA supplementation mimics the gut microbiota alterations associated with ethanol-feeding. Intriguingly, the similarity we identified between the changes introduced by GTA and by ethanol in mice suggests that human studies focusing on alcohol consumption may primarily be revealing effects of acetate, which can also come from numerous other dietary sources, notably starch^45,46^ and/or differences in the microbiome and its metabolism among individuals^47^. Studies aimed at isolating deleterious effects of alcohol on the microbiome or of the alcohol-induced microbiome changes, must carefully detrend for these factors, similar to the well-known initial attribution of signatures to Type 2 diabetes that were ultimately shown to be due to metformin treatment for this condition, requiring careful isolation of variables in follow-up studies^30^. Alcohol consumption frequency was recently identified as a strong factor of microbiota variance between healthy subjects and patients with disease and that can confound study designs^48^. Taken together, our results indicate that acetate might play a key role in the gut microbiota alterations induced by alcohol consumption, and suggest that follow-up studies to explore the effects of the alcohol- and GTA-modified microbiomes into gnotobiotic, treatment-naïve mice may be required to understand the specific functional consequences of alcohol treatment per se on the gut microbiome.

## Methods

All animal studies and research presented here were reviewed and approved by the Institutional Animal Care and Use Committee of the University of California, San Diego or the University of Southern California.

### [1-^13^C] ethanol tracing

C57BL/6 mice (Charles River; females, age 9 weeks) were fed with the Lieber DeCarli diet and the caloric intake from ethanol was 10% on days 1–3, 20% on days 4–6, 30% on days 7–9 and 36% from day 10 until the end of the study period. At day 15, mice were gavaged with [1–^13^ C] ethanol (Sigma; 3 g/kg) in the evening, followed by twice daily gavages on days 16 and 17, and one final gavage in the morning of the day of harvesting (day 18). A subset of mice received ND646 (DC chemical; 50 mg/kg) or vehicle gavage 30 min following each ethanol gavage. Plasma, cecal contents and liver were isolated and total fatty acids were extracted using a Bligh and Dyer-based extraction with methanol, chloroform, and water. Specifically, 500 µl methanol, 200 µl Milli-Q water, and 500 µl chloroform were added to weighed tissue. Samples were vortexed for 5 min followed by centrifugation for 5 min at 18,000 g. Polar metabolites were derivatized in 2% (w/v) methoxyamine hydrochloride (Thermo Scientific) in pyridine and incubated at 37 °C for 60 min. Samples were then silylated with N-tertbutyldimethylsilyl-N-methyltrifluoroacetamide (MTBSTFA) with 1% tert-butyldimethylchlorosilane (tBDMS) (Regis Technologies) at 37 °C for 30–45 min. Polar derivatives were analyzed by GC–MS using a DB-35MS column (30 m x 0.25 mm i.d. x0.25 μm, Agilent J&W Scientific) installed in an Agilent 7890 A gas chromatograph (GC) interfaced with an Agilent 5975 C mass spectrometer (MS). The lower chloroform phase was dried and derivatized through transesterification of fatty acids to form fatty acid methyl esters (FAMEs). Derivatization proceeded with addition of 500 µl 2% H_2_SO_4_ in methanol and incubation for 2 h. FAMEs were then extracted with 100 µl saturated salt solution and 500 µl hexane. The hexane layer (top) consisting of FAMEs was evaporately concentrated and re-solubilized in 100 µl hexane for GCMS analysis. Samples were measured on a FAME select column 100 m x 0.25 mm i.d.) installed in an Agilent 7890 A GC interfaced with an Agilent 5975 C MS using the following temperature program: 80 °C initial, increase by 20 °C/min to 170 °C, increase by 1 °C/min to 204 °C, then 20 °C/min to 250 °C and hold for 10 min.

Isotopomer spectral analysis (ISA)^49,50^ was conducted using the mass isotopomer distribution of palmitate to quantify the contribution of administered [1–^13^ C] ethanol to lipogenic acetyl-CoA pools in each sample. The ISA model estimates two parameters: contribution of the tracer to the precursor lipogenic acetyl-CoA pool and fraction of palmitate newly synthesized from tracer in the measured pool. The INCA metabolic flux analysis software^51^ estimates these parameters through comparison of the measured mass isotopomer distribution of palmitate to a simulated distribution from a model reaction network of palmitate synthesis, which is structured as 8 acetyl-CoA molecules are condensed to form one palmitate molecule.

### Ex vivo experiments

Cecal content from C57BL/6 mice (Charles River; females, age 9 weeks) fed ethanol-containing Lieber DeCarli diet or isocaloric diet for 8 weeks were harvested^52^. Cecal contents were incubated in anoxic basal medium (NH4Cl 0.5 g/L, NaCl 0.4 g/L, KCl 0.05 g/L, KH2PO4 0.05 g/L, MgSO4.7H2O 0.1 g/L, NaHCO3 1 g/L, 100x DL minerals, vitamins, yeast extract 0.05 g/L) containing ethanol, at 37 °C for 120 h. Anaerobic production of SCFAs were measured by high-performance liquid chromatography (HPLC).

### Measurement of serum short chain fatty acids

As previously described, C57BL/6 (Charles River; females, age 9 weeks) were fed Lieber DeCarli diet for 8 weeks to investigate the impact of ethanol intake on serum short chain fatty acid concentrations. The female mice were nine weeks old when the experiment started and were maintained on a 12 h artificial light/dark cycles, a temperature range of 68–72 F, and a humidity of 40–70% RH. After an 8-week feeding period, mice were sacrificed, and blood samples were collected. Plasma samples in 100 ul aliquots were sent to the University of Michigan metabolomics core. Samples were analyzed for short chain fatty acids (SCFAs) using a modified version of a previously described protocol^53^. 20 µL of plasma or media was aliquoted to an Eppendorf tube and 60 µL of extraction solvent (Acetonitrile containing internal standards) was added. Samples were centrifuged at 16,000 xg at 4 °C for 5 min and 40 µL of supernatant was transferred to a 1.8 mL glass autosampler vial. To this vial were added 6 µL of 200 mM 3-nitrophenylhydrazine (3-NPH) in 1:1 acetonitrile:water and 6 µL of 120 mM of 1-Ethyl-3-(3-dimethylaminopropyl)carbodiimide in 1:1 acetonitrile water with 6% pyridine. Samples were capped, vortexed, and placed in a warming oven at 40 °C for 30 min. Once derivatization was complete the samples were cooled and diluted by addition of 348 µL of 90/10 water/acetonitrile; the samples were re-capped and submitted to LC-MS analysis. A standard curve was prepared identically to the samples, substituting 40 uL volatile fatty acid mix (Sigma CRM46975) for samples (and all other extraction/derivatization volumes double), and finally diluted to concentrations ranging from 3 µM to 3000 µM. Samples were analyzed using an Agilent (Santa Clara, CA) 1290 LC coupled to an Agilent 6490 triple quadrupole MS. The chromatographic column was a Waters (Milford, MA) HSS T3, 2.1 mm × 100 mm, 1.7 µm particle size. Mobile phase A was 0.1% formic acid in water; mobile phase B was 0.1% formic acid in methanol. The gradient was as follows: linear ramp from 15% to 80% B from 0–12 min; step to 100% B from 12–12.1 min; hold 100%B from 12.1–16 min; step to 15%B from 16-16.1 min; hold 15%B from 16.1–20 min. The injection volume was 5 µL and the column temperature was 55 °C. MS parameters were as follows: gas temp 325 °C, gas flow 10 L/min, nebulizer 40 psi, capillary voltage 4000 V, scan type MRM, negative ion mode, delta EMV 600. MRM parameters were as indicated in Supplementary Table 3. Quantitation was performed using Agilent MassHunter Quantitative Analysis software version 8.0 by measuring the ratio of peak area of the 3-NPH derivatized SCFA species to its closest internal standard (by retention time). Linear standard curves were used to estimate SCFA concentrations in the extract, which were normalized to the measured mass of cecal contents.

### GTA supplementation

GTA supplementation was performed in an intragastric feeding model of continuous ethanol infusion in C57BL/6 (Jackson) mice as described previously^54,55^. Male mice, eight weeks old when the experiment started, were maintained on 12 h artificial light/dark cycle, a temperature range of 65–75 F, and a humidity 30–70%. First, a small (*N* = 4) dosage trial at GTA feeding at low (0.1 g/kg body weight), median (1.0 g/kg body weight), and high (6.0 g/kg body weight) doses was performed in a dose step up over 9 days with a dose step up every three days. At the end of each dosage treatment blood samples were collected and acetate levels were measures as previously described. In the subsequent experiment, briefly, after one-week acclimatization period with infusion of a control high fat diet through a surgically implanted long-term gastrostomy catheter, GTA or glycerol was added to the diet to achieve the median dose (1.0 g/kg body weight) at the beginning of ethanol-feeding. Diet infusion rate was 400 mL/kg/day. At the initial ethanol dose, total caloric intake is set at 533 Cal/kg and the caloric percentages of ethanol, dietary carbohydrate (dextrose), protein (lactalbumin hydrolysate) and fat (corn oil) are 29%, 13%, 23%, and 35%, respectively, as described. Following seven days of intragastric alcohol feeding, mice were harvested, and cecum, liver, and blood samples were collected for further analysis.

### Biochemical assays and histological analysis of liver tissues

Hepatic injury and steatosis were assessed by serum ALT level, Haemotoxylin and Eosin (H&E) staining and hepatic triglyceride levels^54,56^. Absorption and hepatic metabolism of alcohol in mice was assessed by measuring the blood ethanol level, hepatic ADH activity (BioVision) and microsomal CYP2E1 protein expression by immunoblotting through Anti-Cytochrome P450 Enzyme CYP2E1 Antibody (Millipore)^57^. Fecal albumin was measured by ELISA (Bethyl Laboratories, Montgomery, TX) according to the manufacturer’s instructions.

### Cultivation of *Bacteroides fragilis* with Acetate

To prevent condensation, a clear flat bottom 96-well plate lid was coated with 3 mL of an aqueous solution with 20% ethanol and 0.01% Triton X-100 (Sigma, cat # X100-100ML) in the biosafety cabinet. Excess liquid was removed after 30 s and the lid was allowed to air-dry for 30 min in the biosafety cabinet under a UV light for sterilization. The rest of the steps were conducted in the anaerobic chamber. A culture of *B. fragilis* was grown from a glycerol stock for 16 h before 1 ml of culture was pelleted and resuspended in 1 mL of Clostridium Basal media (CBM)^38^ with no carbon source. In a flat bottom, clear 96-well plate, 10uL of this *B. fragilis* resuspension was then grown in 90ul of CBM + 0.2% glucose, CBM + 20 mM acetate, CBM + 20mM L-methionine, CBM + 20 mM glycerol, CBM + 20 mM acetate + 20mM L-methionine, and CBM + 20 mM acetate + 20 mM glycerol in six replicates. The plate was incubated at 37 °C without shaking in the anaerobic chamber. Optical density readings at 600 nm to monitor for bacterial growth were taken with a Molecular Devices SpectraMax M3 Multi-Mode Microplate Reader (VWR, cat # 89429-536).

### Shotgun metagenomic sequencing and data analysis

Total genomic DNA was extracted using MoBio PowerFecal DNA isolation kit (MoBio) following the manufacturer’s instructions. Purified DNA from all biological replicates per diet group was prepared for shotgun metagenomic sequencing using the Nextera XT library preparation method with average fragment size of 300 base pairs (bp) (Illumina). Libraries were quality assessed using qPCR and a Bioanalyzer (Agilent Technologies) and subsequently sequenced using MiSeq 2 × 300 bp cycle paired-end kit (Illumina). An average of three million non-mouse reads were generated per library.

Raw paired-end reads from the MiSeq platform were initially trimmed and filtered with Trimmomatic (v. 0.39)^58^. Adapter trimmed reads were host-filtered with Bowtie2 (v. 2.3.2)^59^. Paired-end reads were then merged using FLASH (v. 1.2.11)^60^ and co-assembled by the treatment group using metaSPAdes (v. 3.13.1) with kmer length 21, 33, 55, 77, 99, and 127^61^. The coverage depth across all contigs was calculated by aligning raw reads from each sample against the co-assembled contigs using Bowtie2 (v. 2.3.2). The resulting coverage depth was used to bin metagenomic contigs into draft genomes with MetaBAT (v. 2.12.1)^62^, MaxBin (v. 2.2.6)^63^, and CONCOCT (v. 1.0.0)^64^. MetaWRAP (v. 1.2.1)^65^ was used to consolidate multiple binning methods into a final optimal set of draft genomes. CheckM (v. 1.0.3) was used to estimate the contamination and completeness of each draft genome^66^. The draft bacterial genomes were annotated using PROKKA (v. 1.12)^67^. The abundance of each draft genome in each sample was generated using Salmon (v. 0.13.1)^68^. The taxonomic classification of draft genomes was performed through MetaWRAP (v. 1.2.1) with MegaBLAST (v. 2.2.28)^69^ and taxator-tk (v. 1.3.3)^70^. Phylogenetic analysis of the draft genomes was performed through phylophlan (v. 3.0.2) within the phylum level with the set of bins within that phylum by lowest common ancestor^71^, subsequent species level similarity between draft genomes and closest known genomes was compared by orthologous average nucleotide identity^72^.

### Metatranscriptomic sequencing and data analysis

Total RNA was extracted using RNA PowerSoil Total RNA Isolation Kit following the manufacturer’s instructions. Purified RNA was treated with DNAse I Turbo (Invitrogen) and ribosomal RNA was depleted using Ribozero Epidemiology Kit (Illumina). Total cDNA was synthesized using SuperScript IV Kit (Invitrogen). The libraries were prepared for sequencing using the Nextera XT library preparation method with the average fragment size of 200 base pairs (bp) (Illumina). Libraries were quality assessed using qPCR and a Bioanalyzer (Agilent Technologies) and subsequently sequenced using MiSeq 35 bp cycle single-end kit (Illumina). An average of three million non-mouse reads were generated per library.

Metatranscriptomic reads were adapter trimmed and quality filtered with Trimmomatic (v. 0.39). Next, host and rRNA reads were filtered using Bowtie2 (v. 2.3.2), based on the SILVA rRNA databases for bacterial, archaeal, and eukaryotic sequences^73^. The non-rRNA reads were then aligned back to the assembled metagenomic contigs and ORF counts were calculated with Salmon (v. 0.13.1). For both metagenomic bin and metatranscriptomic ORF count tables, the reads mapping count was normalized per kilobase (RPK). The pathway abundance and expression were calculated by the average values for each reaction in the pathway. In the case of multi-copy genes, the most abundant or highly expressed gene was selected.

### Microbiome Statistical analysis

Dimensionality reduction of the metagenomic and metatranscriptomic count tables was performed through Robust Aitchison PCA (DEICODE v. 0.2.4). The resulting ordinations were visualized through matplotlib with samples representing dots and feature loadings representing arrows^74^. The highly weighted features belonging to the phyla Firmicutes and Bacteroides were compared through a log-ratio of the counts through Qurro (v. 0.7.1)^9,75^, with statistical significance evaluated through a two-sided t-test through SciPy (v. 1.4.1)^76^. Similarly, for comparisons of reaction expression or abundance, a log-ratio of counts was compared using the housekeeping gene rpoA as the reference frame^77–79^.

### Genome-scale metabolic modeling of *B. fragilis*

A previously constructed genome-scale metabolic model of *B. fragilis* strain 638 R was used to predict the effects of acetate on the growth of this organism. A GEM contains all of the known metabolic reactions, transporters, and their associated enzymes and metabolites within an organism, as well as a biomass objective representing the metabolites that must be produced for the organism to grow. Additional “exchange reactions”, which represent the availability of individual metabolites in the media, may be activated or inactivated in the model to simulate different in silico conditions. The genome of *B. fragilis* 638 R (Genbank accession number FQ312004) was compared to representative sequences in the KEGG and Transport Classification databases to identify protein functions and reconstruct the metabolic network in this GEM.

To predict the effects of acetate on growth, the GEM was simulated with a basal medium of vitamins, minerals, ammonia, hydrogen sulfide, and inorganic phosphate. One at a time, the exchange reactions for 96 carbon containing metabolites were activated. After simulating the growth rate in each condition, the exchange reaction for acetate was also activated and the simulation ran again. The exchange reactions were given a maximal influx rate of 100 mmol C/gDW/hr. Each condition was run using the COBRA Toolbox in Matlab and the model’s growth rate recorded. Simulated growth rates less than 0.001 hr^−1^ were deemed to represent no growth. Acetate was considered to have had an effect on a given condition if its presence increased the growth rate by at least 0.0001 hr^−1^.

### Statistics & reproducibility

No power analyses or other calculations were used to predetermine sample sizes rather sample sizes were chosen based on prior literature using similar experimental paradigms. The experimental data are reported in full, and the number of mice studied, or number of biological observations studied for each experiment are provided in the methods. Mice of similar age and weight were randomly assigned to experimental and control groups, mice were randomly assigned to experimental and control groups. We had no specific methods to blind the investigators during the experiments, but all mice were treated equally at the same time.

### Reporting summary

Further information on research design is available in the Nature Research Reporting Summary linked to this article.

## Supplementary information

Supplementary Information

Reporting Summary

Supplementary Data 1

## Data Availability

All sequence data and linked sample information can be found in EBI under project ERP138370 (https://www.ebi.ac.uk/ena/browser/view/PRJEB53563) and are available in Qiita study ID 13052. Additional details on accession codes for sequences used in this study are provided in Supplementary Data 1. Source data are provided with this paper.

## Source data

Source Data

## Code availability

All analyses for this study have been deposited on Zenodo (10.5281/zenodo.6807813)^80^.
