# Supplementary material for: Acetate reprograms gut microbiota during alcohol consumption
Source: Nat Commun. 2022 Aug 8;13:4630. doi: 10.1038/s41467-022-31973-2 (PMC9359997; doi:10.1038/s41467-022-31973-2)
Supplement: Supplementary file 1 — Supplementary Information [file 41467_2022_31973_MOESM1_ESM.pdf]

Supplementary Information: Acetate reprograms gut microbiota during alcohol consumption

C. Martino, L.S. Zaramela, B. Gao et al.

Contents

Supplementary Figures 1-7
(*on following pages of this PDF*)

Supplementary Table 1-3
(*on following pages of this PDF*)

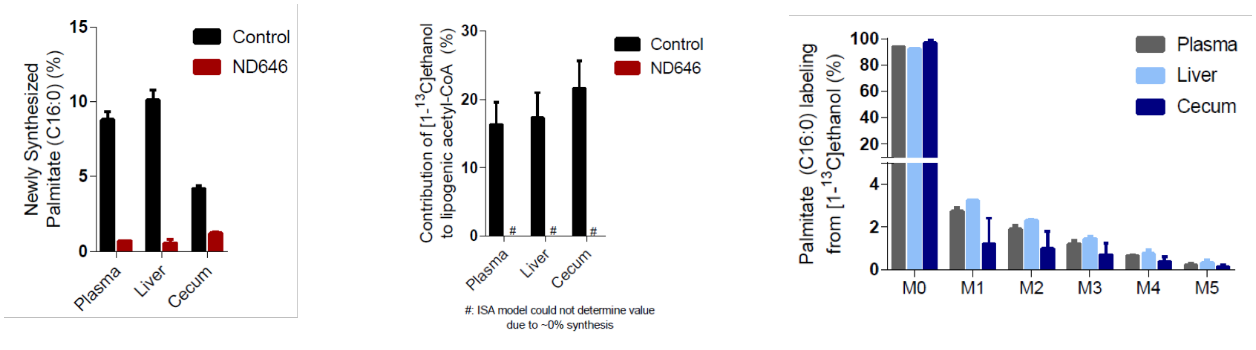

Supplementary Figure 1. De novo lipogenesis from [1-13C] EtOH. 9%, 10%, and 5% palmitate synthesized from 13C ethanol in plasma, liver, and cecum, respectively ACC inhibitor ND646 shut down palmitate synthesis to approximately ~0.5% in plasma, liver, and cecum 13C ethanol contributed to 16%, 17%, and 22% of the lipogenic acetyl-CoA pool in plasma, liver, and cecum, respectively. Bar plots represent the mean value and the error bars the standard error (N=3). Source data are provided as a Source Data file.

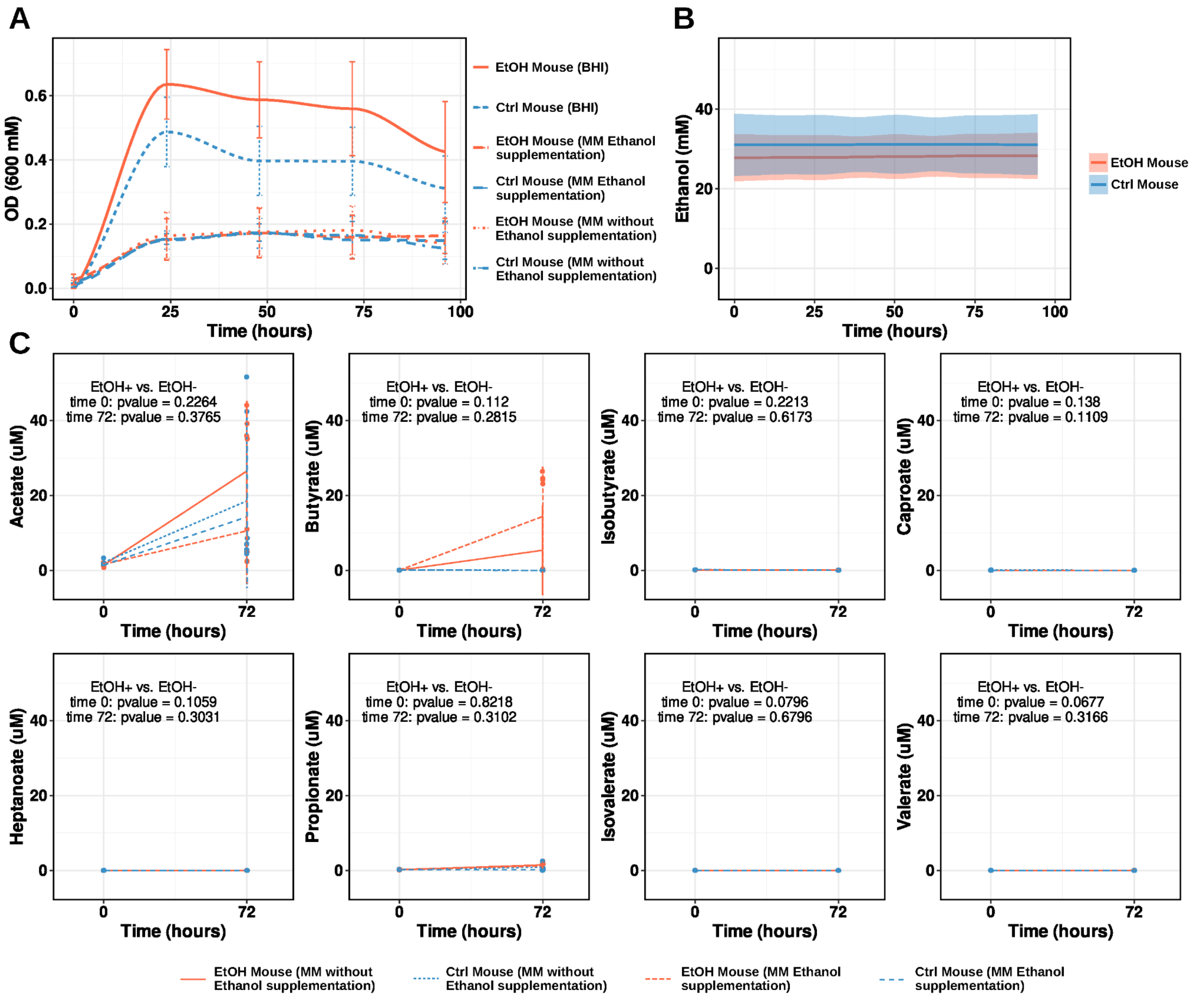

Supplementary Figure 2. Cultivation of whole microbial communities in non-toxic levels of ethanol from mouse cecum content treated with alcohol reveals no anaerobic assimilation of ethanol. (a) Mice fed ethanol (green) and those not (blue) for cecum inoculant and grown culture with ethanol as the sole carbon source, compared cecum grown on Brain Heart Infusion Broth medium (BHI) and Minimal medium (MM) across cultivation growth (hours; x-axes) measured by optical density at 600 nm (y-axis) (N=4 for each condition). (b) Ethanol concentrations measured from cultures grown with or without ethanol as a carbon source. (c) Short Chain Fatty Acid concentrations measured from cultures grown with or without ethanol as a carbon source (x-axis) from cecum from mice fed ethanol (left-panels) and those not (right-panels). Significance was evaluated by a two-sided t-test. The line plots represent the mean value and the error bars the standard error across replicates. Source data are provided as a Source Data file.

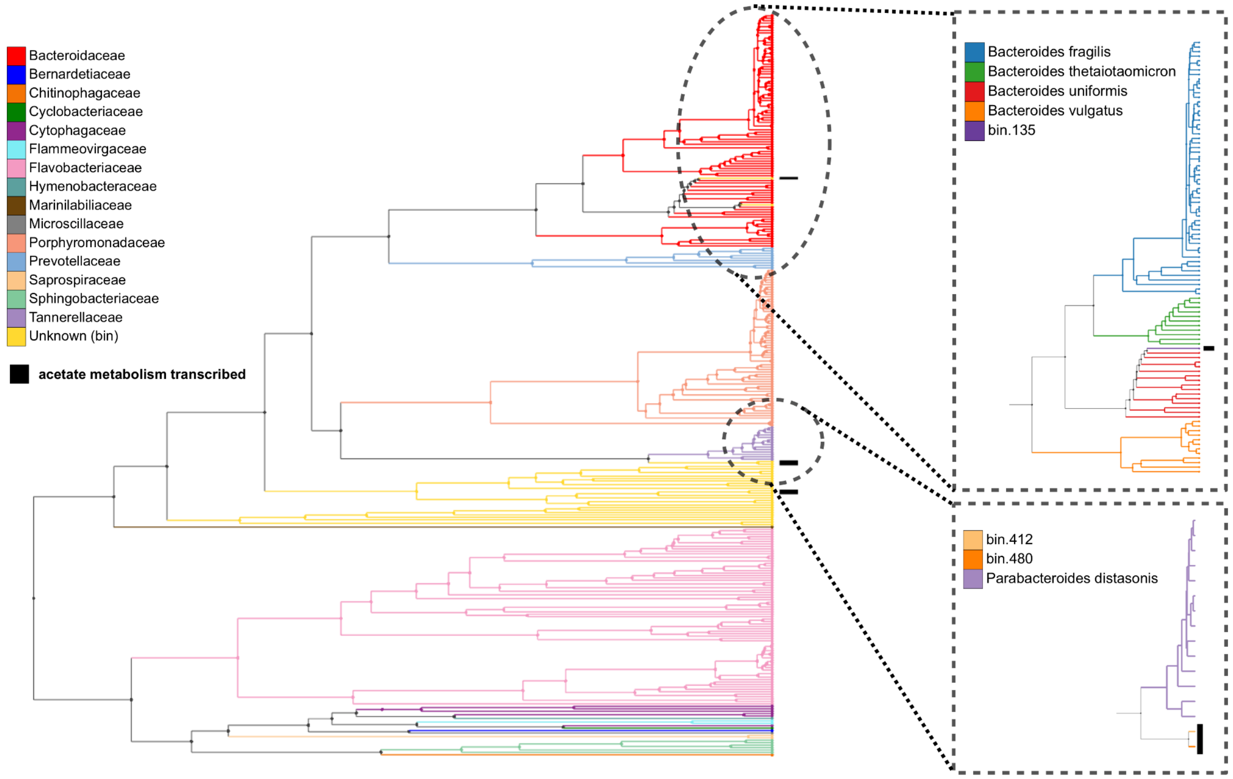

Supplementary Figure 3. (left) Phylogeny of those bins predicted to be in the Bacteroidetes phylum based on the lowest common ancestor of contigs predicted taxonomy, with all completed GenBank genomes within the phylum Bacteroidetes colored by family level taxonomy, black bar annotation indicated those bins containing and transcribing acetate metabolism genes (E.C. 6.2.1.1 and 1.2.1.10). (right) Sheared tree zoomed in on those bins with completed reference bins within the constructed phylogenetic clade, colored by the species level taxonomy.

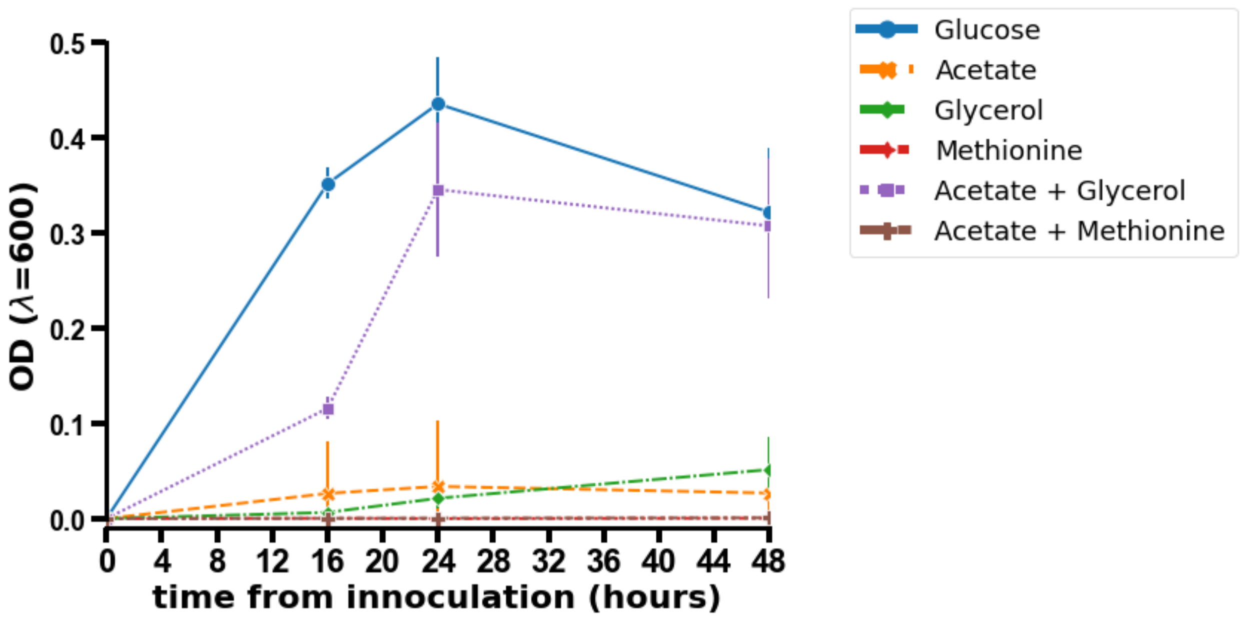

Supplementary Figure 4. *Bacteroides fragilis* cultured with glucose or acetate with or without co-metabolism substrates methionine or glycerol for 48 hours with growth (x-axis) of the culture across time measured through optical density at 600 nm (y-axis). Negative control cultures and blanks were subtracted from the conditions. The line plots represent the mean value and the error bars the standard error across replicates. Source data are provided as a Source Data file.

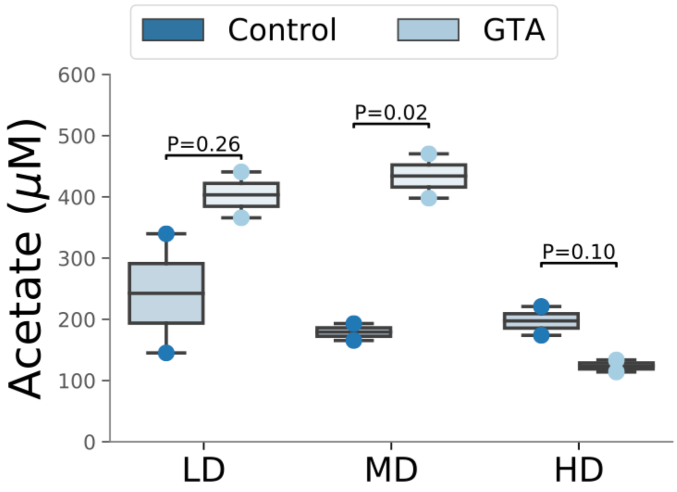

Supplementary Figure 5. Serum acetate levels (y-axis) compared to controls across doses of GTA feeding at low (0.1g/kg body weight), medium (1.0g/kg body weight), and high (6.0g/kg body weight) doses (x-axis) (N=4). A feeding model over 9 days with a dose step up every 3 days was used. Significance was evaluated by a two-sided t-test. Box plots represent the minimum, maximum, median, first, and third quartile values (shaded region). Source data are provided as a Source Data file.

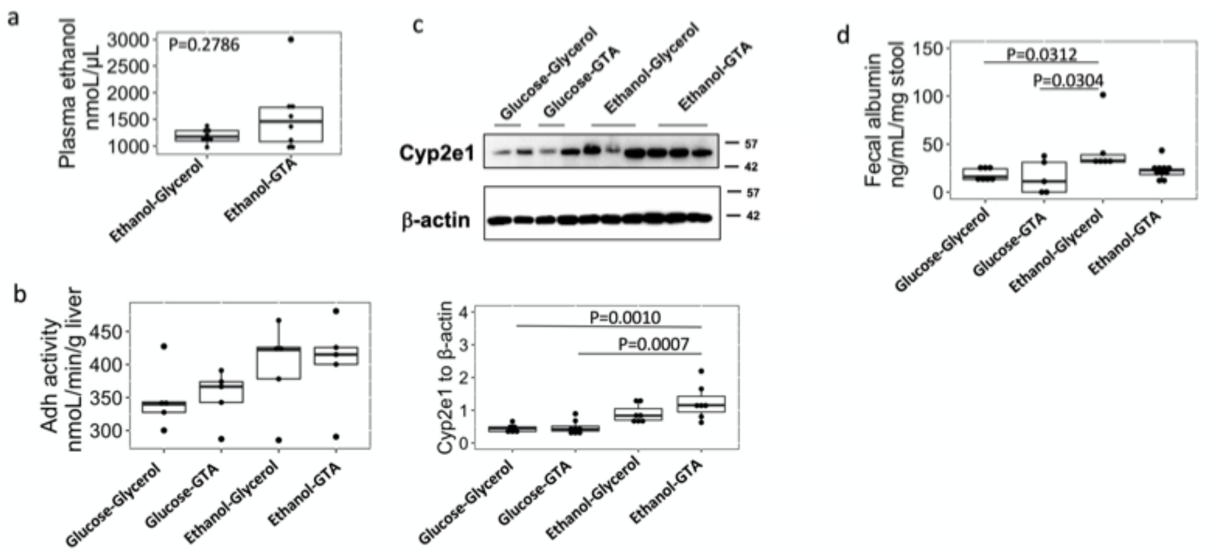

Supplementary Figure 6. Absorption and hepatic metabolism of alcohol, and intestinal permeability assessment. (a) Plasma ethanol level was not significantly different between two groups. Two-sided Wilcoxon rank sum test p-value=0.2786. (b) Hepatic alcohol dehydrogenase (Adh) activity was not significantly altered between different groups. Two-way ANOVA test on alcohol effect p-value = 0.0767, GTA effect p-value = 0.8300, and interaction p-value = 0.9673. (c) Immunoblotting of microsomal Cyp2e1 protein expression. Two-way ANOVA test on alcohol effect p-value = 5.21E-05, GTA effect p-value = 0.130, and interaction p-value = 0.212. (d) Fecal albumin levels. Two-way ANOVA test on alcohol effect p-value = 0.0376, GTA effect p-value = 0.0365, and interaction p-value = 0.1216. Tukey's HSD post-hoc test adjusted p-values less than 0.05 were shown in the figure. Box plots represent the minimum, maximum, median, first, and third quartile values (shaded region) (N=29). Source data and uncropped blots are provided as a Source Data file.

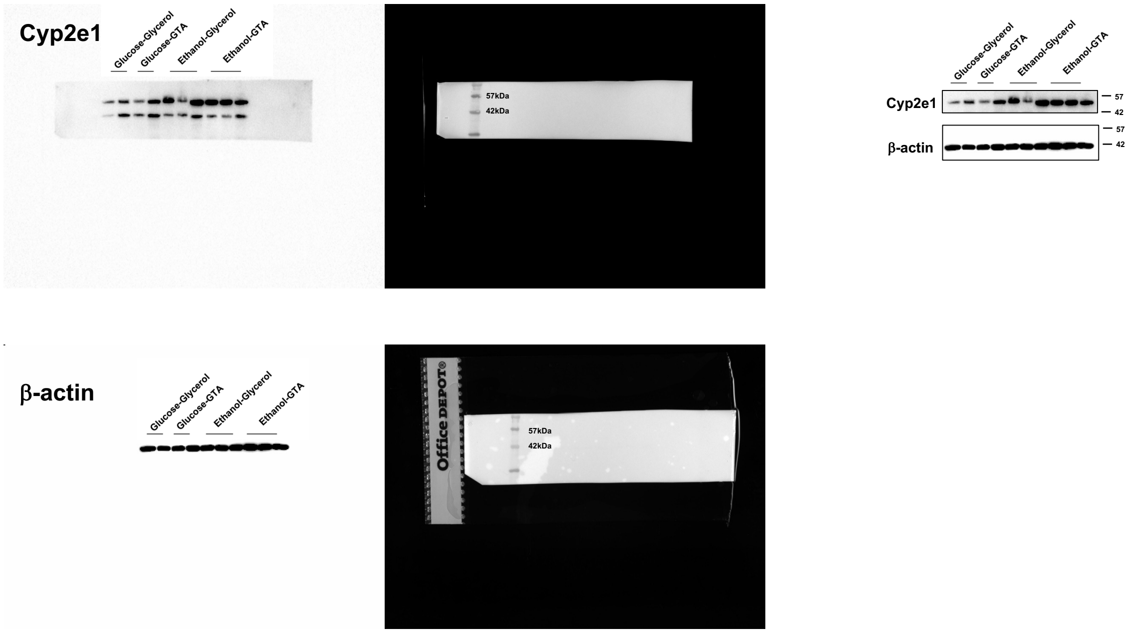

Supplementary Figure 7. Uncropped and unprocessed immunoblots and the markers for microsomal Cyp2e1 protein expression.

Supplemental Tables

Supplementary Table 1. Free-energy changes at pH=7 were calculated from Delta G^0 values provided in *Thauer et al.* [31]. Synthesis of ATP (from ADP) or phosphate bonds requires 32 kJ/mol and 22kJ/mol respectively [31]. Thus, neither the anaerobic oxidation of ethanol nor of acetate provides sufficient energy to support life.

		Substrate (reactants)	Product	Delta G0 (kJ/mol)
**ethanol oxidation**	**Anaerobic**	$C_2H_8O_2 + H_2O$	$C_2H_3O_2^- + 5H^+ + 4e^-$	1
	**Aerobic**	$C_2H_8O_2 + O_2$	$C_2H_3O_2^- + H^+ + 2H_2O$	-730.8
**acetate oxidation**	**Anaerobic**	$C_2H_3O_2^- + H^+ + 2H_2O$	$2CO_2 + 8H^+ + 8e^-$	201.9
	**Aerobic**	$C_2H_3O_2^- + H^+ + O_2$	$2CO_2 + 4H^+ + 4e^-$	-551.1

Supplementary Table 2. Those bins containing and transcribing acetate metabolism genes (E.C. 6.2.1.1 and 1.2.1.10) with their closest relative by phylogenetic distance compared by average nucleotide identity (ANI).

**Binned Genome ID (genome A)**	**bin.135**	**bin.480**	**bin.412**
**Reference Strain (genome B)**	Bacteroides uniformis 2789STDY5834898	Parabacteroides distasonis FDAARGOS 344	Parabacteroides distasonis FDAARGOS 345
**BioProject**	PRJEB10915	PRJNA231221	PRJNA231222
**BioSample**	SAMEA3545361	SAMN06173357	SAMN06173358
**Genbank accession**	GCA_001405595.1	GCA_002206325.2	GCA_002206325.3
**RefSeq accession**	GCF_001405595.1	GCF_002206325.1	GCF_002206325.2
**Reference Source**	feces, human	feces, human	feces, human
**ANI value (%)**	91.97	73.99	74.32
**Genome A length (bp)**	3,169,140	5,365,200	5,457,000
**Genome B length (bp)**	4,919,460	5,111,220	5,111,220
**Average aligned length (bp)**	1,463,993	1,370,617	1,346,666
**Genome A coverage (%)**	46.2	25.55	24.68
**Genome B coverage (%)**	29.76	26.82	26.35

Supplementary Table 3. MRM scan parameters for LC-MS/MS SCFA assay.

Compound name	ISDT	Precursor Ion	MS1 Res	Production	MS2 Res	Dwel	Fragmentor	Collision Energy	Cell accelerator Voltage	Polarity
C7-3NPH		264.1	Unit	137.1	Unit	75	325	19	7	Negative
C6-3NPH_D11	X	261.1	Unit	137.1	Unit	75	325	19	7	Negative
C6-3NPH		250.1	Unit	137.1	Unit	75	325	19	7	Negative
C5-3NPH		236.1	Unit	137.1	Unit	75	325	19	7	Negative
C5-3NPH_D7	X	229.1	Unit	137.1	Unit	75	325	19	7	Negative
C4-3NPH		222.1	Unit	137.1	Unit	75	325	19	7	Negative
C3-3NPH		208.1	Unit	137.1	Unit	75	325	19	7	Negative
C2-3NPH_D3	X	197.1	Unit	137.1	Unit	75	325	19	7	Negative
C2-3NPH		194.1	Unit	137.1	Unit	75	325	19	7	Negative
