# Supplementary material for: Acetate reprograms gut microbiota during alcohol consumption
Source: Nat Commun. 2022 Aug 8;13:4630. doi: 10.1038/s41467-022-31973-2 (PMC9359997; doi:10.1038/s41467-022-31973-2)
Supplement: Supplementary file 2 — Reporting Summary [file 41467_2022_31973_MOESM2_ESM.pdf]

nature research

Corresponding author(s): Karsten Zengler

Last updated by author(s): Jul 2, 2022

Reporting Summary

Nature Research wishes to improve the reproducibility of the work that we publish. This form provides structure for consistency and transparency in reporting. For further information on Nature Research policies, see our Editorial Policies and the Editorial Policy Checklist.

Statistics

For all statistical analyses, confirm that the following items are present in the figure legend, table legend, main text, or Methods section.

n/a	Confirmed	
☐	☒	The exact sample size (*n*) for each experimental group/condition, given as a discrete number and unit of measurement
☐	☒	A statement on whether measurements were taken from distinct samples or whether the same sample was measured repeatedly
☐	☒	The statistical test(s) used AND whether they are one- or two-sided<br>*Only common tests should be described solely by name; describe more complex techniques in the Methods section.*
☐	☒	A description of all covariates tested
☐	☒	A description of any assumptions or corrections, such as tests of normality and adjustment for multiple comparisons
☐	☒	A full description of the statistical parameters including central tendency (e.g. means) or other basic estimates (e.g. regression coefficient) AND variation (e.g. standard deviation) or associated estimates of uncertainty (e.g. confidence intervals)
☐	☒	For null hypothesis testing, the test statistic (e.g. *F*, *t*, *r*) with confidence intervals, effect sizes, degrees of freedom and *P* value noted<br>*Give P values as exact values whenever suitable.*
☒	☐	For Bayesian analysis, information on the choice of priors and Markov chain Monte Carlo settings
☒	☐	For hierarchical and complex designs, identification of the appropriate level for tests and full reporting of outcomes
☐	☒	Estimates of effect sizes (e.g. Cohen's *d*, Pearson's *r*), indicating how they were calculated

Our web collection on statistics for biologists contains articles on many of the points above.

Software and code

Policy information about availability of computer code

Data collection	Sequencing was performed at the UC San Diego Institute for Genomic Medicine Genomics Facility using the Illumina MiSeq sequencing instrument. Sequencing data supporting the findings of this study will be available upon acceptance.
Data analysis	Trimmomatic (v. 0.39), FLASH (v. 1.2.11), metaSPAdes (v. 3.13.1), MetaBAT (v. 2.12.1), MaxBin (v. 2.2.6), CONCOCT (v. 1.0.0), MetaWRAP (v. 1.2.1), CheckM (v. 1.0.3), PROKKA (v. 1.12), Salmon (v. 0.13.1), MetaWRAP (v. 1.2.1), MegaBLAST (v. 2.2.28), taxator-tk (v. 1.3.3), Bowtie2 (v. 2.3.2), Qurro (v. 0.7.1), SciPy (v. 1.4.1), DEICODE (v. 0.2.4), and deblur (v. 1.1.0). Python scripts to generate figure and for statistical analysis are available at https://github.com/cameronmartino/acetate-alcohol.

For manuscripts utilizing custom algorithms or software that are central to the research but not yet described in published literature, software must be made available to editors and reviewers. We strongly encourage code deposition in a community repository (e.g. GitHub). See the Nature Research guidelines for submitting code & software for further information.

Data

Policy information about availability of data

All manuscripts must include a data availability statement. This statement should provide the following information, where applicable:

- Accession codes, unique identifiers, or web links for publicly available datasets
- A list of figures that have associated raw data
- A description of any restrictions on data availability

All sequence data and deidentified participant responses can be found in EBI under project PRJEB53563 and Qiita study ID 13052.

Field-specific reporting

Please select the one below that is the best fit for your research. If you are not sure, read the appropriate sections before making your selection.

☒ Life sciences ☐ Behavioural & social sciences ☐ Ecological, evolutionary & environmental sciences

For a reference copy of the document with all sections, see nature.com/documents/nr-reporting-summary-flat.pdf

Life sciences study design

All studies must disclose on these points even when the disclosure is negative.

Sample size	No power analyses or other calculations were used to predetermine sample sizes. Sample sizes were chosen based on prior literature using similar experimental paradigms (Nature Communications. 2017;8:2137; Nature. 2019;575:505-1511)
Data exclusions	No data exclusions. For the experiments, the data are reported in full.
Replication	The number of mice studied, or number of biological observations studied for each experiment are provided in the methods
Randomization	Mice of similar age and weight were randomly assigned to experimental and control groups.
Blinding	Mice were randomly assigned to experimental and control groups. We had no specific methods to blind the investigators during the experiments, but all mice were treated equally at the same time.

Reporting for specific materials, systems and methods

We require information from authors about some types of materials, experimental systems and methods used in many studies. Here, indicate whether each material, system or method listed is relevant to your study. If you are not sure if a list item applies to your research, read the appropriate section before selecting a response.

Materials & experimental systems

n/a	Involved in the study	
☒	☐	Antibodies
☒	☐	Eukaryotic cell lines
☒	☐	Palaeontology and archaeology
☐	☒	Animals and other organisms
☒	☐	Human research participants
☒	☐	Clinical data
☒	☐	Dual use research of concern

Methods

n/a	Involved in the study	
☒	☐	ChIP-seq
☒	☐	Flow cytometry
☒	☐	MRI-based neuroimaging

Animals and other organisms

Policy information about studies involving animals; ARRIVE guidelines recommended for reporting animal research

Laboratory animals	GTA supplementation: C57BL/6 (Jackson) male mice, 8 weeks old when the experiment started [1-13C] ethanol tracing and ex vivo experiments: C57BL/6 (Charles River) female mice, 9 weeks old when the experiment started. [1-13C] ethanol tracing and ex vivo experiments mice were maintained on 12h artificial light and dark cycle. Temperature range is 68-72F and the Humidity 40-70% RH. GTA supplementation mice were maintained on 12h artificial light and dark cycle. Temperature range is 65-75F; Humidity 30-70%.
Wild animals	The study did not involve wild animals.
Field-collected samples	The study did not involve samples collected from the field.
Ethics oversight	All animal studies were reviewed and approved by the Institutional Animal Care and Use Committee of the University of California, San Diego or the University of Southern California.

Note that full information on the approval of the study protocol must also be provided in the manuscript.
